# Novel Insights into the Therapeutic Potential of Curcumin and Derivatives

**DOI:** 10.3390/ijms24108837

**Published:** 2023-05-16

**Authors:** Beatrice E. Bachmeier

**Affiliations:** Institute of Pharmaceutical Biology, Goethe University Frankfurt, 60438 Frankfurt am Main, Germany; b.bachmeier@em.uni-frankfurt.de

The polyphenol curcumin (diferuloylmethane) is extracted from the plant turmeric (*Curcuma longa*), and it is widely used as a spice component or coloring agent. The antioxidant compound has been applied in traditional Indian and Chinese medicine to treat inflammatory disorders. In Ayurveda (traditional Indian medicine), turmeric powder has been used for various purposes and through different routes of administration, such as topically for wounds, blistering diseases, parasitic skin infections, and acne. Orally, it has been administered for the common cold, liver diseases, urinary tract diseases, and as a blood purifier. Even against chronic diseases, such as rhinitis and coryza, it has been used via inhalation [1,2]. All these examples show the anti-inflammatory properties of curcumin. More recently, the anti-tumorigenic [3] and anti-metastatic effects [4] of curcumin, as well as its therapeutic potential in neurodegenerative diseases, have become the focus of research [5]. This is not surprising, as the development of neoplastic and neurodegenerative diseases, as well as brain injury are connected to inflammatory components inasmuch the same signal transduction pathways are activated intracellularly. Although many preclinical studies have been performed in the last three decades to delineate the molecular mechanisms of curcumin, there are still unanswered questions, which will be in part addresses by the 19 papers published in this Special Issue.

The lipophilic character of curcumin along with the problems of drug transport and metabolism leading to the paradigm of low concentration in the bloodstream despite its therapeutic effects are subjects of debate. Three papers in this issue address this interesting research topic. A good explanation for the discrepancy between the clinical benefits and the low serum concentrations of curcumin could be that polyphenol acts directly in the gut, having a beneficial effect on the microflora, as postulated in the review by Pluta and colleagues [6]. Therefore, curcumin possibly eliminates intestinal microflora dysbiosis, which has recently been associated with the development of neurodegenerative, neurological, and cancer diseases. As a possible mode of action, the authors suggest that curcumin undergoes enzymatic modifications by bacteria in the gut, forming pharmacologically more active metabolites than the parental compound. Cardoso’s research group tackles the problem of curcumin’s transport and release [7]. They developed a very small magnetic nanocarrier exhibiting superparamagnetic properties with a crystalline structure of less than 10 nm. These particles—carrying curcumin—can be magnetically guided to the site where its therapeutic action is required (e.g., tumors), and in addition, drug release can be combined with magnetic hyperthermia. Last but not least, Castano and co-workers investigated the inhibitory effects of curcumin on steroid metabolism and tested the effect of curcuminoids on cytochromes P450, and in particular, on the enzymes CYP17A1, CYP21A2, and CYP19A1. The results of this work suggest that curcuminoids with modulated molecular structure should serve as lead compounds for the drug design of effective CYP17A1 and CYP19A1 inhibitors [8].

The importance of novel therapy strategies including plant-derived pharmacologically effective compounds, such as curcumin, in the treatment of neurodegenerative disorders becomes clear as 6 of the 19 papers address this topic [9,10,11,12,13,14]. Three of these are original papers on the effects of curcumin or derivatives in Parkinsons’s disease (PD). El Nebrisi and co-workers describe that curcumin acts neuroprotectively inasmuch it significantly improves the abnormal motoric behavior linked to (PD) via an a7-nicotinic acetylcholine receptor-mediated mechanism [9]. Another paper indicating the beneficial effects of curcumin on PD has been provided by Buratta et al. [10]. The group shows that curcumin and its antioxidative properties abolish protein damage, including the formation of carbonylated and nitrotyrosine-derived proteins caused by the pesticide rotenone. Wang and co-workers investigated the effect of a curcumin derivative on protection against PD [11]. In their studies, the curcumin derivative activated the transcription factor EB (TFEB), which is considered a new therapeutic target of PD. After activation, TFEB translocates into the nucleus. This phenomenon is accompanied by enhanced autophagy and lysosomal biogenesis. Therefore, the compound promotes the degeneration of a-synuclein and protectes against the cytotoxicity of 1-methy-4-phenylpropidium ion in neuronal cells in vitro.

In addition to the three original papers on this interesting topic, three reviews have been published on the effects of curcumin or its derivatives against neurodegeneration. The review provided by Panaro and co-workers summarizes what has been published so far on the neuroprotective and anti-rheumatic properties, and delineates in particular the modulation of the Toll-like-receptor-4 (TLR-4) signaling pathway, which plays a crucial role in immune response and the production/stimulation of inflammatory cytokines [12]. Another excellent overview on the clinical efficacy of curcumin analogues and its derivatives in Alzheimer’s disease (AD), as well as their use in diagnostic applications is provided by Chainoglou and co-workers [13]. They summarize the current knowledge on how these compounds and, in particular, their phenyl methoxy groups contribute to the anti-amyloidogenic activity. In addition, their review points out the role of curcumin-based compounds with their keto-enol tautomerism as being useful for diagnostic imaging in AD. Another very interesting review is provided by Ułamek-Kozioł and co-workers [14] who assess the therapeutic benefits of curcumin during the development of neurodegeneration after brain ischemia and explain that curcumin can even penetrate the blood–brain barrier and neuronal membranes. In this review, the authors summarize curcumin’s pleotropic properties, including its anti-amyloid, anti-inflammatory, anti-apoptotic, and neuroprotective effects.

We know from traditional medicine and many preclinical studies that curcumin has anti-inflammatory properties, which come into effect when it inhibits inflammation-associated signaling pathways, and therefore, it is not surprising that its therapeutic potential against inflammation-associated diseases is currently eagerly explored. In this context, Wu and colleagues [15] showed that curcumin inhibited the NFkB, MAPK, AKT and pBAD pathways in vivo in two different mouse models representing acute kidney injury and chronic kidney disease. Curcumin’s potential to abrogate the production of inflammatory cytokines and chemokines has already been a research subject in the context of breast cancer [16,17], and in the current Special Issue, Vitali and colleagues correlate this mode of action to the development of the Herpes Simplex Virus Type 2 (HSV-2) infection [18]. Their results demonstrate that curcumin is delivered into the vaginal tract and reduces local tissue inflammation, and this could potentially reduce the severity of HSV-2 infection, as well as the risk of HIV acquisition. Finally, a review summarizes the potential of curcumin for the treatment of endometriosis by acting on inflammation, oxidative stress, invasion, adhesion, apoptosis, and angiogenesis [19]. These results, together with previously published data, underline that curcumin has therapeutic potential for the treatment of inflammatory and inflammation-associated diseases.

Last but not least, the anti-tumorigenic effects of curcumin are always an extremely interesting research topic. In this Special Issue, the influence of curcumin on triple-negative breast cancer, gastrointestinal cancer, glioblastoma, hepatocellular carcinoma, bladder cancer, colorectal cancer, and cancer cell metabolism in general, has been dealt with in seven of the 19 papers. Modi and co-workers described the synergistic effects of a curcumin derivative in combination with Paclitaxel in triple-negative breast cancer cells in vitro [20], although the toxicity and bioavailability of this synthesized compound is still to be elucidated. Glioblastoma is an extremely aggressive and hard-to-treat brain tumor. Despite treatment by radiation and chemotherapy after the surgical elimination of the tumor, patients have an extremely bad prognosis with survival rates of less than one year. Therefore, any promising treatment option is a bright spot, as is the paper of Wang and co-workers, validating the enhancing effects of curcumin on radiotherapy in glioblastoma-bearing rats [21]. Here, they elucidate that curcumin in combination with radiotherapy increases overall survival periods most probably by causing a G2/M cell cycle arrest. In another paper, the effect of curcumin and its solvent ethanol on cell metabolism, in particular on glucose uptake and lactate production, was examined. However, while curcumin modulated the cell metabolism, the solvent ethanol also showed significant effects [22]. The effects of curcumin on DNA damage induced by Benzo(a)pyrene (BaP), a carcinogen formed during cooking, was examined in stomach tissue of Sprague Dawley rats by Kim and colleagues [23]. Here, curcumin significantly reduced formation of damaged DNA adducts in the livers, kidneys, and stomachs of the rats. The effects of curcumin as a photosensitizer in the treatment of cancer cells have already been described [24]. In this issue, this effect has been described in the context of chronic viral hepatitis and hepatocellular carcinoma [25]. Furthermore, Rutz and colleagues summarize the therapeutical effects of curcumin as a chemosensitizer and a complementary treatment agent for bladder cancer [26], and Pricci and coworkers point out the therapeutic potential of curcumin for the treatment of colorectal cancer [27].

In conclusion, this Special Issue highlights novel insights into molecular mechanisms underlying the therapeutic benefits of curcumin and its derivatives for many applications. Although curcumin derivatives seem to exhibit better bioavailability than the lipophilic polyphenol itself, their safety is still to be explored, while curcumin—although less bioavailable—shows promising effects in combination with chemo- and radiotherapy.

As curcumin itself is very well tolerated, it would be worth to continue testing it clinically in well-designed treatment strategies for selected ailments. In addition, a number of nanocarrier formulations of curcumin have been evaluated as “safe” and clinically beneficial [28]. Additionally, the market for nutraceutical products is expanding, in particular for enriched turmeric supplements, which became a top-selling product in the United States. On the other hand, it is still difficult to draw a final conclusion on the clinical efficacy of curcumin in humans, despite the fact that many clinical trials have been performed so far. Reasons for this could be that, among other things, dose-finding or pharmacokinetic data are scarce and studies on the comparative effects of different products are missing. This makes it difficult to translate the promising results of preclinical studies into therapeutic applications. Clinical studies in the future should be designed with great care and diligence, and should include the above-mentioned aspects in order to be able to draw better conclusions on the medical benefits of curcumin.
